# Quantitative Gait Analysis Using Inertial Sensors: The Influence of Sensor Configuration on Extracted Gait Parameters

**DOI:** 10.3390/jfmk11040433

**Published:** 2026-10-19

**Authors:** Marco Antonio Lira-Zamacona, Alma Delhi De León-Hernández, Carlos Omar López-López, Adriana Martínez-Hernández

**Affiliations:** Instituto de Investigación Aplicada y Tecnología, Universidad Iberoamericana Ciudad de México, Mexico City 01219, Mexico; marcolira317@aragon.unam.mx (M.A.L.-Z.); alma.deleon@ibero.mx (A.D.D.L.-H.); carlos.lopez@ibero.mx (C.O.L.-L.)

**Keywords:** gait analysis, inertial measurement units, wearable sensors, gait kinematics, spatiotemporal parameters

## Abstract

Inertial Measurement Units (IMUs) have emerged as a practical alternative for quantitative gait analysis. Although numerous studies have demonstrated their capability to estimate spatiotemporal and kinematic gait parameters, there is no clear consensus regarding the relationship between sensor configuration and the gait information that can be extracted. This review aimed to examine the relationship between IMU configuration, including sensor number and anatomical placement, and the quantitative gait parameters obtained, while also considering the processing methods used to derive these parameters. A structured literature review was conducted using PubMed, ScienceDirect, and Web of Science databases. Articles published between 2014 and 2025 were screened according to predefined inclusion and exclusion criteria. Information regarding sensor quantity, sensor placement, extracted gait parameters, processing methods, and validation systems was analyzed. A total of 59 studies were included. Sensor configurations ranged from one to seventeen IMUs. The extracted gait parameters were classified into gait phases, spatiotemporal parameters, and joint kinematics. The findings indicate that the relationship between the number of sensors and the gait parameters obtained is not linear. Sensor placement and signal-processing methods were found to be as important as sensor quantity in determining the gait parameters obtained. The number of IMUs alone is not a reliable predictor of the amount of gait information that can be extracted. Instead, the combination of sensor placement and processing methodology plays a critical role in determining the gait parameters obtained. These findings suggest that IMU configurations should be selected according to the gait parameters of interest rather than by maximizing the number of sensors employed.

## 1. Introduction

Gait is a fundamental movement pattern that humans perform, enabling us to move from one place to another and carry out activities of daily living. For this reason, its assessment has attracted significant interest in the clinical field, particularly in fields such as rehabilitation, orthopedics, and geriatrics, for patient diagnosis and monitoring [1,2,3]. Gait is characterized and analyzed by understanding its spatiotemporal parameters (step length, stride length, step width, foot angle, speed, cadence, and cycle phases) and its kinematic parameters (joint ranges of motion) [4].

Currently, gait assessment in clinical practice is performed using observational tests such as the “Timed Up and Go” and the “Short Physical Performance Battery,” which can determine gait abnormalities based on the time taken to complete the tasks and the observation of the gait pattern; this observation may be influenced by the experience of the evaluators, which can lead to variations in results between observers [5,6].

Several technological tools are available for quantitative gait analysis, such as video-based optical systems (e.g., VICON, Oxford, United Kingdom), which are considered the gold standard for human motion analysis, and instrumented platforms (e.g., GAITRite, NJ, USA) for analyzing spatial and temporal gait parameters. However, these systems require trained personnel and controlled environments specifically designed for their use; furthermore, they are expensive, making them inaccessible to most hospitals or clinical centers, particularly in developing countries [7].

Recently, new technologies for human motion analysis have emerged, such as systems based on Inertial Measurement Units (IMUs) [8], which have already been validated against optical systems and have demonstrated a high correlation with them [9,10]. Due to their characteristics, such as small size, portability, and the fact that they do not require external infrastructure such as cameras, platforms, or instrumented mats to accurately track human movement, IMUs represent a viable alternative for gait analysis [11,12]. Furthermore, thanks to these characteristics, IMUs can be used to assess movement outside the hospital setting, such as on the street, at home, or in work areas, allowing evaluation of movement during daily activities and contributing to greater ecological validity [13]. However, their application requires the implementation of complex algorithms to estimate the orientation of each body part where a sensor is placed to obtain the body’s joint ranges of motion [14].

Specifically, gait assessment using IMUs has enabled the acquisition of kinematic and spatiotemporal gait parameters. Most studies have successfully detected the gait phases (stance and swing phases), and, based on this segmentation, it becomes easier to obtain other parameters, such as cadence, step length, and stride length [15,16]. On the other hand, some studies have focused on joint kinematics to determine joint ranges of motion during the gait cycle [17].

Several reviews have previously examined the use of inertial sensors for gait and lower-limb kinematic analysis. Petraglia et al. [18], Kobsar et al. [19], and more recently, Prisco et al. [20] primarily evaluated the validity and reliability of IMU-derived measurements compared with laboratory-based reference systems. Weygers et al. [21] focused specifically on methodological approaches for estimating lower-limb joint kinematics from inertial sensors, whereas Caldas et al. [22] reviewed inertial-sensor-based gait analysis methods and the adaptive algorithms used for gait assessment. Prasanth et al. [23] focused on real-time detection methods and their application to pathological gait, whereas Lin et al. [24] found that gyroscopes remain the primary tool for detecting gait events, while using the full IMU (gyroscope + accelerometer) improves reliability and enables estimation of spatiotemporal parameters. In contrast, reviews such as Mobbs et al. [25] have analyzed only the clinical viability of single-IMU systems (single-point placement) on the lower trunk. More recently, García-de-Villa et al. [8] provided a comprehensive review of inertial-sensor-based human motion analysis, considering aspects such as processing algorithms, number of sensors, and body regions instrumented.

Despite these contributions, previous reviews have not specifically addressed the relationship between IMU configuration and the quantitative gait parameters that can be extracted as a central research question. In particular, it remains unclear how the number and placement of IMUs relate to the ability to obtain gait phases and events, spatiotemporal parameters, and lower-limb joint kinematics, and how the processing methods used contribute to these outcomes. Addressing this gap is relevant for determining whether complex multi-sensor configurations necessarily provide additional clinically meaningful gait information or whether strategically selected sensor configurations can provide similar information with reduced instrumentation.

Therefore, this narrative state-of-the-art review examines the relationship between IMU configuration, particularly the number and anatomical placement of sensors, and the quantitative gait parameters obtained. In addition, the processing methods used to derive these parameters and the reference systems employed for validation are examined to provide a broader understanding of the methodological factors that determine the information obtainable from IMU-based gait analysis.

## 2. Materials and Methods

This study was designed as a narrative state-of-the-art review rather than as a systematic review. The objective was to identify and analyze technological trends regarding IMU sensor configurations, sensor placement, and signal-processing methods used for quantitative gait analysis, rather than to synthesize clinical evidence or evaluate the methodological quality of the included studies. However, the literature search and study selection process followed the general principles of the PRISMA flow chart.

The literature review process consisted of five stages. Figure 1 presents the flowchart of the article selection process.

In stage 1, an advanced search was conducted in PubMed, ScienceDirect (on 29 July 2026 for both databases), and Web of Science (on 3 August 2026) using combinations of keywords and MeSH (Medical Subject Headings) terms, along with the Boolean operators AND and OR. Two search strategies were defined:

I. (gait OR “gait analysis”) AND (“gait kinematics” OR “joint angles”) AND (“inertial measurement unit”).

II. (gait OR “gait analysis”) AND (“spatiotemporal parameters”) AND (“inertial measurement unit”).

In stage 2, the selection criteria for the articles were established:

I. Articles published from 1 January 2014 to 31 December 2025 were considered eligible.

II. For ScienceDirect and Web of Science, articles published in journals related to biomechanics, rehabilitation engineering, biomedical instrumentation, sensors, and motion analysis were considered.

III. Articles published in English.

In stage 3, articles were selected based on their title, excluding those that:

I. Included populations with a specific pathology.

II. Implemented artificial intelligence methods.

III. Evaluated upper-limb movements.

IV. Included gait tests under non-natural walking conditions (under fatigue or perturbation conditions).

In stage 4, duplicate articles were identified across both searches and databases.

Finally, in stage 5, following an abstract screening of each article, the following exclusion criteria were applied:

I. Did not extract or report gait parameters.

II. Did not perform gait tests.

III. Evaluated only pathological gait patterns.

IV. Did not compare gait metrics with a gold-standard reference system.

V. Systematic reviews and meta-analyses.

## 3. Results

Using the methodology described, 6790 articles were initially identified. After applying the screening process, inclusion, and exclusion criteria, 59 articles were included in this review. These selected articles implemented IMU sensors to obtain gait parameters, and the results were validated against a reference system. This section generally describes the number of sensors employed, the gait parameters obtained, the algorithms implemented, and the reference systems used to validate the results. Table 1 details the number of sensors, their location on the body, the gait parameters obtained, and the algorithms or methods applied in each selected article.

All data analyzed in this review were extracted from the original publications included in the study. Information regarding sensor configuration and placement, gait parameters, processing methods, and reference systems was obtained directly from the corresponding articles, where the complete methodological and experimental data are available.

### 3.1. Number of Inertial Sensors

Sensor configurations ranged from one to 17 IMUs. Two-sensor configurations were the most frequently reported (15 studies), followed by seven-sensor configurations (12 studies), one-sensor configurations (9 studies), and three-sensor configurations (7 studies). Configurations of 10, 11, 14, and 17 sensors represented the largest systems included in the review.

### 3.2. Gait Parameters

The extracted gait variables were classified as gait phases and events (GP), spatiotemporal parameters (STP), and joint angles (JA). Several studies reported variables belonging to more than one category.

GP comprises relevant events such as Heel Strike (HS) and Toe-off (TO), as well as the stance and swing phases and their subdivisions. Gait phases and events were reported in 25 studies. Spatial parameters include step length, stride length, and step width, while temporal parameters include cadence, step time, and speed. One or more STPs were reported in 27 studies. Finally, joint angles (JA) were reported in 33 studies and include hip, knee, ankle, and, in some studies, pelvic or trunk kinematics.

### 3.3. Algorithms and Processing Methods

This section provides a general description of the algorithms and processing methods used to obtain gait variables, regardless of the number of sensors used in each study. Given the high degree of methodological heterogeneity in inertial signal processing, the algorithms and methods identified in the 59 selected studies were categorized into four fundamental families: Orientation Estimation (OE), Signal Processing (SP), Calibration and Correction (CC), and Model-Based Methods (MB).

#### 3.3.1. Orientation Estimation (OE)

Estimating the 3D orientation of body segments is a fundamental step in most inertial kinematic analyses. The Kalman filter and its variants (e.g., Extended Kalman Filter or Error-State Kalman Filter) were the most frequently reported orientation-estimation approaches, followed by Madgwick, Mahony, and complementary filters. Several studies relied on proprietary orientation-estimation software.

#### 3.3.2. Signal Processing (SP)

Signal processing techniques were primarily applied to temporally segment the gait cycle and derive spatial metrics from raw or oriented data. For gait event detection (Heel Strike and Toe-Off), the predominant methodology involved identifying morphological features in the kinematic signals, such as local maxima/minima, zero-crossings, or peaks in the angular velocity and linear acceleration. More advanced frequency-domain analyses employed Continuous Wavelet Transforms (CWTs) or wavelet decompositions to isolate impact frequencies. Furthermore, to obtain spatial parameters such as stride length or foot trajectory, the standard operation across the literature was the mathematical double integration of gravity-compensated linear acceleration. Some authors also utilized the first derivative of foot Euler angles for event detection.

#### 3.3.3. Calibration and Correction (CC)

Specific calibration and correction strategies were implemented to address sensor misalignment and mitigate accumulated integration errors (drift). To mitigate integration drift during gait, Zero Velocity Updates (ZUPTs) or Zero-Angle Updates (ZAUs) were applied during stationary or quasi-stationary phases as state-correction strategies. Regarding anatomical alignment, Principal Component Analysis (PCA) was applied to functional gait data to align the sensor’s virtual axes with the functional planes of rotation. Advanced sensor-to-segment alignment was also achieved through specific calibration matrices derived from static poses (gravity vectors), functional calibration movements, or cross-product vector operations.

#### 3.3.4. Model-Based Methods (MB)

Model-based approaches explicitly incorporated biomechanical, anatomical, or kinematic assumptions into spatial or kinematic estimation. To bypass the errors associated with double integration, various studies employed the Inverted Pendulum model, which relies on anthropometric segment lengths to estimate spatial displacement analytically. More sophisticated approaches applied inverse kinematics polynomial modeling, biomechanical constraints, or full musculoskeletal models via software like OpenSim v.4.2. These approaches used anatomical or biomechanical constraints to reduce the solution space and support spatial or joint-kinematic estimation.

#### 3.3.5. Proprietary Methods and Reporting Limitations

Finally, a limitation identified in the current literature is the reliance on black-box systems. Several studies utilized completely closed, proprietary algorithms (e.g., Xsens, BTS SMART-Clinic) to extract all parameters without disclosing the underlying signal processing.

### 3.4. Relationship Between Processing Methods and Extracted Gait Parameters

A distinct relationship was observed between the processing-method families and the type of gait information reported by the included studies. Because individual studies could report more than one gait-parameter category and implement multiple processing approaches, the frequencies described below represent study-level co-occurrence and are not mutually exclusive.

Signal Processing (SP) was the predominant methodological family among studies reporting gait phases and events (GP), being identified in 18 of 25 studies (72.0%). In contrast, Orientation Estimation (OE) appeared in 9 studies (36.0%), while Calibration and Correction (CC) and Model-Based Methods (MB) were less frequent, appearing in 4 (16.0%) and 2 studies (8.0%), respectively. These findings indicate that gait-phase and event identification was primarily based on characteristic features of acceleration or angular-velocity signals, including peaks, local minima or maxima, zero-crossings, and wavelet-based methods.

A similar, although less pronounced, pattern was observed for spatiotemporal parameters (STP). SP methods were identified in 16 of the 27 studies reporting STP (59.3%), followed by OE in 12 studies (44.4%). CC and MB methods appeared in 5 studies each (18.5%). This pattern was associated with a broader range of processing approaches for STP, including gait-event segmentation, numerical integration, trajectory reconstruction, and biomechanical models. STP extraction involved a broader range of processing approaches: temporal variables were frequently derived from gait-event segmentation, whereas spatial variables such as step or stride length additionally involved numerical integration, trajectory reconstruction, or biomechanical models.

In contrast, studies reporting joint angles (JA) showed a clear predominance of OE methods. Orientation-estimation approaches were identified in 24 of 33 studies (72.7%), whereas SP and CC were each present in 11 studies (33.3%), and MB approaches were identified in 4 studies (12.1%). The combination of OE and CC occurred in 10 of the 33 studies reporting JA (30.3%), representing the most frequent combined processing pattern associated with joint-kinematic outcomes.

Overall, these results indicate that the gait information obtained from an IMU-based system depends on the interaction between sensor configuration and processing methodology. GP and many STP variables were commonly obtained through signal-processing methods applied to a limited number of strategically positioned sensors, whereas joint kinematics were more strongly associated with orientation-estimation and calibration/correction approaches applied to sensors located on anatomically relevant adjacent segments. Overall, the co-occurrence analysis showed different methodological profiles across gait-parameter categories: GP and STP were more frequently associated with SP methods, whereas JA was more strongly associated with OE and OE+CC approaches.

### 3.5. Reference Validation Systems

This section briefly describes the reference system used to validate the parameters obtained with the inertial sensors for each study. All studies validated IMU-derived measurements against a reference system. Fifty studies reported using video-based systems for validation, with VICON being the most commonly reported (29 studies). While nineteen studies employed force platforms and/or instrumented walkways as reference tools.

## 4. Discussion

This narrative state-of-the-art review analyzed studies published between 2014 and 2025 that implemented inertial measurement units (IMUs) for quantitative gait assessment. The objective was to determine whether a relationship exists between the number of IMUs used and the gait parameters that can be extracted. Previous reviews have demonstrated the validity and reliability of IMU-based gait measurements compared with laboratory-based reference systems [18,19] and have described the methodological approaches used for lower-limb kinematic estimation [21]. The present review complements these findings by showing that the amount and type of gait information obtained cannot be explained solely by the number of sensors employed. Although configurations with a larger number of IMUs generally enable multi-joint kinematic analysis, several studies using one or two strategically placed sensors were able to obtain specific spatiotemporal parameters, gait events, or even single-joint kinematics. This aligns with the findings of Mobbs et al. [25], who confirmed the clinical validity of single-point IMUs for estimating spatiotemporal parameters without the need for complex setups; however, they present significant limitations when attempting to estimate complex metrics of asymmetry and variability. Conversely, some multi-sensor configurations were designed to obtain only a limited set of gait parameters. These findings suggest that sensor placement and processing methodology, rather than sensor count alone, are critical determinants of the information obtainable from an IMU-based gait analysis system. This concept extends the experimental findings of Panebianco et al. [81], who showed that the accuracy and repeatability of gait event detection vary drastically by sensor location—trunk, tibia, or foot—and by the physical variable analyzed (linear acceleration or angular velocity). Overall, when contextualizing our findings within the existing literature, it becomes evident that the added value of an IMU-based gait analysis system lies not in maximizing hardware capabilities, but in the effective synergy between a strategically placed sensor topology and a processing algorithm aligned with clinical objectives.

### 4.1. Relationship Between Sensor Configuration and Extracted Gait Parameters

The principal finding of this review is that the number of IMUs alone cannot predict the amount or type of gait information that can be obtained. Although studies employing more sensors generally reported a broader characterization of gait, several studies demonstrated that sensor placement plays an equally important role.

Studies using a single IMU have primarily focused on extracting spatiotemporal gait parameters and identifying gait phases and events [11,16,26,27,28,29,32]. Several of these investigations placed the sensor in the lumbar or pelvic region and successfully estimated cadence, step and stride times, step and stride lengths, and gait speed. Other studies identified gait events such as heel strike (HS) and toe-off (TO) directly from accelerometer signals or estimated spatiotemporal parameters using the kinematics of a single body segment, such as the thigh (Arumukhom et al. [26]).

Similarly, studies employing two IMUs generally obtained spatiotemporal parameters and detected gait phases and events. The most common placements were on the feet or shanks, allowing the identification of HS and TO events from which cadence, step length, stride length, and temporal gait parameters could be derived. However, several studies demonstrated that two strategically positioned sensors on the thigh and shank can be sufficient to estimate knee joint kinematics [36,38,43,44,45,46]. These findings indicate that the information obtained depends more strongly on the biomechanical relevance of the monitored segments than on the number of sensors alone.

The importance of sensor placement becomes even more evident when comparing studies that employed the same number of sensors. For example, both Niswander et al. [60] and Yeo et al. [61] used five IMUs, but with different anatomical distributions and different outcomes. Niswander et al. focused on gait-phase and event detection using sensors distributed along the tibia and foot, whereas Yeo et al. estimated spatiotemporal parameters and hip and knee kinematics using sensors placed on the shanks, thighs, and pelvis. This contrast illustrates that sensor location and anatomical coverage can substantially influence the information obtained, even when sensor count is identical. A similar trend emerged in studies using seven or more IMUs. Most of these investigations placed sensors on the feet, shanks, thighs, and pelvis, frequently adding sensors on the trunk, chest, or head. Such configurations enabled the estimation of lower-limb joint kinematics, including ankle, knee, and hip joint angles. Nevertheless, some studies employing seven or more sensors focused exclusively on spatiotemporal gait parameters, demonstrating that increasing the number of sensors does not necessarily result in a broader characterization of gait [64,71].

A clear distinction emerged between the extraction of spatiotemporal and kinematic gait parameters. Researchers commonly obtained spatiotemporal parameters by identifying gait events, particularly heel strike and toe-off. Once these events were detected, the signals could be segmented into steps or strides, allowing the estimation of cadence, step time, stride time, step length, and stride length. Most studies achieved this through peak-detection methods applied to accelerometer or gyroscope signals, particularly along the anteroposterior and mediolateral axes. Consequently, extracting spatiotemporal parameters often required only a limited number of sensors.

The contrast between reduced and multi-segment configurations indicates that sensor placement determines the biomechanical information available to the processing algorithm. A sensor placed on the foot or shank provides signals strongly associated with gait-event timing, whereas joint kinematics require measurements from the segments defining the joint. Sensor count should therefore be interpreted as a measure of anatomical coverage rather than as an independent indicator of information content.

### 4.2. Role of Signal Processing in Gait Parameter Extraction

The different methodological profiles identified for GP/STP and JA reflect their different computational requirements. Gait events can frequently be identified from local characteristics of acceleration or angular-velocity signals, whereas joint angles depend on the relative orientation of adjacent anatomical segments.

This distinction explains why processing methodology can, to some extent, compensate for reduced sensor configurations. Event-based approaches can extract clinically useful temporal information from a single strategically positioned sensor, whereas multi-joint kinematics requires increased anatomical coverage. Calibration and correction procedures become particularly relevant in this context because errors in sensor-to-segment alignment directly propagate to joint-angle estimates.

Thus, sensor number, placement, and processing methodology should not be considered independent design variables. Their interaction determines the information obtainable from an IMU system.

### 4.3. Practical Implications and Future Directions

The results of this review have important implications for the design of wearable gait-analysis systems. Increasing the number of sensors may provide more biomechanical information; however, it also increases system complexity, setup time, user burden, and cost. Therefore, selecting an appropriate sensor configuration should balance the required information against the practical constraints of clinical and real-world applications.

The reviewed studies suggest that relatively simple sensor configurations may suffice when the objective is limited to gait event detection or estimating spatiotemporal parameters. Conversely, applications requiring lower-limb joint kinematics require additional sensors on the relevant body segments and robust orientation-estimation algorithms.

Although this review focused on the relationship between sensor configurations and the gait parameters obtained, rather than on measurement accuracy, all included studies validated their results against a reference system. Optical motion-capture systems were the most frequently reported validation method, with Vicon being the most commonly used reference system.

A key area for future research is how sensor placement affects measurement accuracy. Studies such as those by De Jong et al. [39], Trojaniello et al. [32], and Niswander et al. [60] investigated the effect of placing sensors at different locations within the same body segment to identify positions less affected by soft-tissue artifacts. Likewise, several studies explored methods to compensate for misalignment between sensor orientation and anatomical segment orientation, including sensor-to-segment alignment techniques [57,67,76] and functional calibration procedures [38,45,52,67].

Future investigations should continue exploring optimal sensor-placement strategies, alignment methods, and sensor-fusion approaches to improve the accuracy and clinical applicability of IMU-based gait analysis systems while preserving their portability, accessibility, and ease of use.

## 5. Conclusions

This review examined the relationship between IMU sensor configuration and the gait parameters obtained during quantitative gait assessment. The findings show that sensor count alone does not determine the amount or type of gait information that can be extracted. Although larger configurations generally provide greater anatomical coverage, the resulting gait information also depends on sensor placement and the processing methodology applied.

The methodological analysis showed that gait phases and many spatiotemporal parameters were predominantly associated with signal-processing approaches based on event detection and signal features, whereas joint-kinematic outcomes were more frequently associated with orientation-estimation methods and, in several cases, calibration and correction procedures. These findings indicate that IMU configuration should be selected according to the gait outcomes of interest, the anatomical segments that must be instrumented, and the processing strategy required to obtain them.

Increasing the number of sensors may provide additional biomechanical information, but it also increases system complexity, setup time, user burden, and cost. Future research should therefore focus on identifying efficient sensor configurations, improving sensor-to-segment alignment and calibration methods, and developing robust orientation-estimation and processing approaches that preserve the portability and clinical applicability of IMU-based gait-analysis systems.

## Figures and Tables

**Figure 1:**
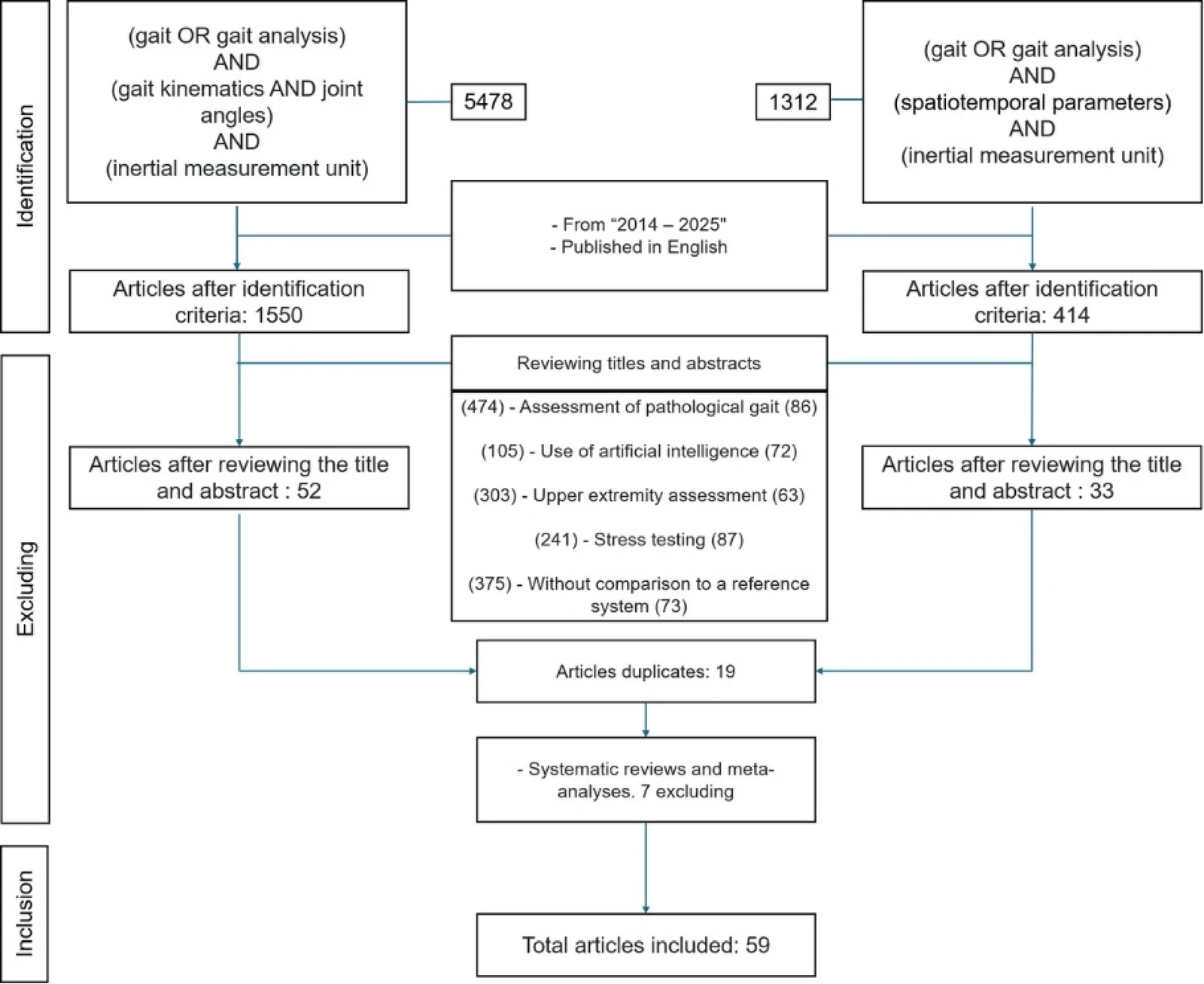
Flowchart of the methodology implemented for the selection of the articles.

**Table 1:** Summary of the selected studies, including the number and brand of IMUs, sensor placement, extracted gait parameters, algorithms or processing methods used to obtain these parameters, and the reference systems used for validation. Gait parameters are classified as gait phases and events (**GP**), spatiotemporal parameters (**STP**), and joint angles (**JA**). Algorithms and Processing Methods are grouped into orientation estimation (**OE**), signal processing (**SP**), calibration and correction (**CC**), and model-based methods (**MB**).

Author	Number of Sensors	Sensors Brand and Their Locations	Extracted Gait Parameters	Algorithms/Processing Methods	Reference System
Arumukhom et al. [26]	1	MTw, Xsens, Enschede, Netherlands.	**STP**: Stride length, stride time, and walking speed.	**Proprietary method**: Quaternion-based thigh orientation obtained using Xsens MT Manager. **SP**: Peak detection of maximum thigh flexion for stride segmentation; polar-radius analysis based on the phase relationship between thigh angle and angular velocity.	**OMoCap**: Qualisys (Gothenburg, Sweden) with 18 cameras.
		Distal third of the thigh (lateral side).			
Lerín-Calvo et al. [16]	1	BAIOBIT, BTS Bioengineering, Milan, Italy.	**GP**: Stance phase, swing phase, and single-leg stance phase. **STP**: Cadence, walking speed, stride length, and step length.	**Proprietary method**: BTS SMART-Clinic algorithms.	**OMoCap**: SMART-DX 6000 (BTS Bioengineering) with 8 cameras.
		Second sacral vertebra (S2).			
Zamberger et al. [11]	1	BTS-G-Walk system, BTS Bioengineering, Milan, Italy.	**GP**: Times of stance and swing phases, and double-support and single-support phases. **STP**: Cadence, walking speed, stride length, and stride time.	**Proprietary method**: BTS-GStudio algorithms.	**OMoCap**: VICON with 16 cameras.
		Pelvis (L5-S1).			
Zampier et al. [27]	1	Trigno Avanti, Delsys, MA, USA.	**GP**: HS and TO events.	**SP**: Peak detection (maximum/minimum) on raw data accelerometer derivatives.	**OMoCap**: Qualisys with 12 cameras + 2 force platforms (BMS400600 AMTI, MA, USA).
		Sacrum.			
Ráfales-Perucha et al. [28]	1	RunScribe system, CA, USA.	**GP**: Ground contact time and swing time. **STP**: Cadence, step time, step length, stride length, and stride time.	**Proprietary method**: RunScribe algorithms to obtain metrics.	OptoGait Optoelectronic System (Microgate, Bolzano, Italia).
		Right foot dorsum.			
Kitagawa et al. [29]	1	TSND121, ATR-Promotions, Kyoto, Japan.	**GP**: Swing phase. **STP**: Stride length.	**SP:** Double Integration of gravity-compensated acceleration data during the swing phase. **CC:** ZUPT (zero-velocity updates).	**OMoCap**: MAC3D System (CA, USA).
		Foot dorsum.			
Bolink et al. [30]	1	MicroStrain Inertia-Link, ND, USA.	**JA**: Pelvis (in sagittal and frontal planes).	**Proprietary method**: Proprietary sensor fusion algorithm for joint angle estimation.	**OMoCap**: Vicon MX-3 cameras and MX-T20 2 cameras + Kistler (Winterthur, Switzerland) 9281A pressure plate.
		Pelvis (between both posterior superior iliac spines).			
Strick et al. [31]	1	Not reported.	**GP**: HS and TO events.	**SP:** Local minimum of thigh angle for HS event; 4-rule classification model for TO event.	Pressure-sensing insoles.
		Thigh.			
Trojaniello et al. [32]	1	Opal, APDM, OR, USA.	**GP**: Stance time, swing time, and double support time. **STP**: Stride time and step time.	**SP:** Method Z: anteroposterior acceleration data minimum to obtain HS; Method G: anteroposterior acceleration data maxima to obtain HS; Method M: Gaussian Continuous Wavelet Transform (CWT) on vertical acceleration data to obtain HS; Method S: sliding window sum and zero-crossings to obtain the step timing; Method K: wavelet-based decomposition of vertical acceleration to obtain HS.	**OMoCap**: Vicon T20 6 cameras + 2 AMTI (MA, USA) force platforms.
		S2 (method Z) L3 (method G) L5 (method M) Waist posterior (method S) Right side waist (method k)			
Anwary et al. [33]	2	MetaWearCPro sensor, CA, USA.	**GP**: Ground contact time and swing time. **STP**: Stride length, step width, distance traveled, step time, stride time, cadence, stride speed, and step speed.	**OE**: Quaternion-based algorithm using the Madgwick method. **SP**: Local minima/maxima and abrupt transitions of the signal; double trapezoidal integration to obtain the length.	**OMoCap**: Qualisys with 18 cameras + force platforms.
		Both feet at the base of the first metatarsal.			
He et al. [34]	2	Foot Secret, Shanqi Wisdom Medical Technology, Chongqing, China.	**GP**: Percentage of stance and swing phases. **STP**: Stride length, cadence, and speed.	**Not Reported**: Algorithms not specified by authors.	**OMoCap**: Qualisys.
		Heels.			
Jocham et al. [35]	2	PABLO Motion Sensor, Tyromotion GmbH, Graz, Austria.	**GP**: Stance phase time and swing phase time. **JA**: Foot orientation (vertical lift, lateral displacement, angular trajectories in the 3 planes).	**OE:** Using quaternions, with yaw determined without a magnetometer. **SP:** Angular velocities and accelerations to detect HS and TO events. **CC:** The orientation and horizontal position determine the foot’s angle of progression.	**OMoCap**: VICON with 16 cameras.
		Forefoot (both feet).			
Allseits (A) et al. [36]	2	Opal, APDM.	**GP**: Gait cycle time. **JA**: Knee (flexion/extension).	**SP:** Angular velocity integration; Noise-Zero Crossing (NZC) algorithm. **CC:** A Zero-Angle Update (ZAU) was performed at the zero-crossing of the angular velocity to correct for drift.	**OMoCap**: VICON with 16 cameras.
		Shin and thigh (anterior).			
Salminen et al. [37]	2	Blue Trident, Vicon, Oxford, United Kingdom.	**GP**: HS, TO, heel lift, feet adjacent, and vertical tibia.	**SP**: CSAV (Cumulative Shank Angular Velocity) zero-crossings; post-negative-cross local minima.	**OMoCap**: Vicon 11 cameras. AMTI force plates.
		Shins (above the lateral malleolus).			
McGrath et al. [38]	2	Opal, APDM.	**JA**: Knee (flexion/extension).	**OE:** Kalman filter. **CC**: Principal Component Analysis (PCA) for axis alignment.	**OMoCap**: Vicon 14-camera system.
		Tibia (lateral) and thigh (anterior).			
De Jong et al. [39]	2	APDM system.	**GP**: HS and TO events. **JA**: Tibial angle relative to the vertical.	**Proprietary method**: Quaternion-based software for obtaining orientation. **SP**: Angular velocity thresholds to detect HS and TO.	**OMoCap**: Vicon 10 cameras + force plates from Kistler Instruments.
		Tibia (anterior and lateral).			
Kluge et al. [40]	2	Shimmer 3, Dublin, Ireland.	**GP**: Support phase time and swing phase time, HS and TO events. **STP**: Stride length, step time, and speed.	**OE**: Euston Complementary Filter. **SP**: Identification of maximum acceleration, angular velocity zero-crossing, and minimum vector magnitude; double integration using the Zak method. **CC**: Position signal smoothing using a sigmoid function.	**OMoCap**: Simi Reality Motion Systems (Unterschleißheim, Germany) with 8 cameras
		Ankles (lateral, below the malleolus).			
Carroll et al. [41]	2	Physilog 5 Gait Up Motion Sensor, Lausanne, Switzerland.	**STP**: Stride time, stride length, and velocity.	**Proprietary method:** Gait Up algorithms to obtain metrics.	GAITRite, NJ, USA.
		In the shoes, below the lateral malleolus.			
Reinhardt et al. [42]	2	RehaGait, Magdeburg, Germany.	**GP**: Stance time, single support time, and double support time. **STP**: Stride length and cadence.	**Proprietary method:** RehaGait algorithms to obtain metrics.	Gaitway 3D treadmill (Hasomed, Magdeburg, Germany).
		In the shoes, below the lateral malleolus.			
Sousse et al. [43]	2	DyCare, Barcelona, Spain.	**JA**: Knee (flexion/extension).	**OE**: Obtained using the Madgwick algorithm.	**OMoCap**: Proreflex Qualisys 8 cameras.
		Thighs (external lateral) and tibia (external lateral).			
Robert-Lachine et al. [44]	2	MTw, Xsens.	**JA**: Knee (flexion/extension, adduction/abduction, and external/internal rotation).	**Proprietary method**: orientation obtained using a quaternion-based algorithm. **SP**: X-Y’-Z’’ Cardan angle sequence.	**OMoCap**: Emovi KneeKG system (QC, Canada).
		Right thigh above the knee axis of rotation (external lateral) and shin (anteromedial lateral).			
Patel et al. [45]	2	i-Sens, Seoul, South Korea.	**STP**: Stride time, stride length, cadence, and speed. **JA**: Hip (flexion/extension) and knee (flexion/extension).	**OE**: Complementary Algorithm. **CC**: Static calibration (offset elimination).	**OMoCap**: Vicon 12 cameras.
		Thigh (external lateral) and shin (lateral tibial condyle).			
Bijalwan et al. [46]	2	BWT 61CL-E, Shenzhen, China.	**JA**: Joint trajectories of the ankle, knee, and hip.	**OE**: Embedded Kalman filter. **SP**: Double integration to obtain position.	**OMoCap**: Microsoft Kinect V2 (WA, USA) with 2 cameras.
		Thigh (near the hip), shin (above the ankle) of the right leg. Three different positions change on hip, knee and ankle of the left and right leg.			
Rudisch et al. [47]	2 and 3	GaitUp Mobility Lab, APDM.	**GP**: Gait cycle time, stance phase, swing phase, single limb support, and double limb support. **STP**: Stride length, cadence and speed.	**Proprietary method:** GaitUp & Mobility Lab embedded algorithms to obtain metrics.	**OMoCap**: OptoGait optoelectronic system + GAITRite mat + Zebris FDM (Isny im Allgäu, Germany) pressure walkway.
		Both feet (dorsum). Both feet (dorsum) and lower back (L5).			
Kvist et al. [48]	3	Opal, APDM.	**GP**: Single support, double support, HS and TO events. **STP**: stride width, step time, cadence, and speed.	**OE**: Madgwick method. **SP**: Continuous Wavelet Transform on medio-lateral gyroscope to detect HS and TO. **MB**: Inverted Pendulum Model to calculate gait phase.	**OMoCap**: Vicon 12 cameras.
		Feet (instep) and lumbar region.			
Johnston et al. [49]	3	Shimmer3.	**STP**: Step time and HS event.	**SP**: The lumbar IMU was used for maximum acceleration and minimum velocity.	AMTI force platforms.
		Tibial (lateral) and lumbar (L3).			
Oliveira et al. [50]	3	MTw, Xsens.	**JA**: Knee and hip (flexion/extension for both joints).	**Proprietary method**: Orientation obtained using an Xsens algorithm. **SP**: Joint angles were calculated by subtracting the distal segment from the proximal segment.	**OMoCap**: Qualisys with 6 cameras.
		Right tibia (posterior), right femur (anterior), and sacrum.			
Sy et al. [51]	3	MTw, Xsens.	**STP**: Stride length, walking speed, and total distance covered. **JA**: Knee (flexion/extension) and hip (internal/external rotation, abduction/adduction, and flexion/extension).	**Proprietary method**: Orientation obtained using an Xsens algorithm. **SP**: Extended Kalman Filter predicting the positions of the ankle and pelvis. **MB**: Applying IMU-based distance constraints and biomechanical constraints (Lie Group).	**OMoCap**: Vicon.
		Shins (above the ankle) and pelvis.			
Nazarahari (A) et al. [52]	3	MTw, Xsens.	**JA**: Ankle and knee (flexion/extension, abduction/adduction, and internal/external rotation for both joints).	**Proprietary method**: Orientation obtained via a proprietary algorithm. **SP**: Principal Component Analysis (PCA) is applied to the sagittal plane velocity, assuming a quasi-monoaxial rotation. **CC**: A vertical calibration method was employed, and that orientation matrix was used.	**OMoCap**: Vicon 8 cameras.
		Thigh (external lateral), shin (anteromedial), and foot (dorsum).			
Horenstein et al. [53]	3	Opal, ADPM.	**JA**: Hip (flexion/extension, abduction/adduction and internal/external rotation).	**OE**: Causal Kalman filter and state-space model (Quaternions via APDM software v3.2.0). **CC**: Time-varying rotation matrices (function “quatrotate” in MATLAB version 9.5).	**OMoCap**: Vicon 10 cameras.
		Sacrum and thighs (external lateral).			
Okkalidis et al. [54]	4	MPU-9250, InvenSense, Taiwan.	**STP**: Foot trajectory (foot clearance) and segment velocities.	**OE:** The Madgwick filter was implemented. **SP**: Zero velocity identification (Gyroscope signals < 30°/s for 0.1 s) to obtain HS. **CC**: Resetting of positioning error accumulation with Zero Velocity Updates ZUPT.	**OMoCap**: Vicon 8 cameras.
		Tibia (posterior), heel, feet (dorsum), and toe (hallux).			
Hafer et al. [55]	4	Opal v2, ADPM.	**JA**: Pelvis (tilt), ankle, knee, and hip (flexion/extension for all 3 joints).	**OE**: Kalman filter (Quaternions). **SP**: The stride was segmented based on the vertical acceleration from the foot sensor using a 1D Continuous Wavelet Transform (CWT).	**OMoCap**: Vicon 15 cameras + AMTI 2 force plates
		Sacrum, lateral thigh, lateral shin, and feet (dorsum).			
Washabaugh et al. [56]	4	Opal, APDM.	**GP**: Stance percent, swing percent, and gait cycle time. **STP**: Velocity, stride length, cadence, and step time.	**Proprietary method**: Algorithms of the APDM Mobility Lab v1 software to obtain metrics.	Bertec instrumented split-belt treadmill (OH, USA).
		Feet (dorsum) and ankles.			
Nazarahari (B) et al. [57]	4	MTws, Xsens.	**JA**: Ankle, knee, and hip (flexion/extension, abduction/adduction, and internal/external rotation for all 3 joints).	**Proprietary method**: Orientations from the system itself. **CC**: Combined static-functional calibration for sensor-to-segment alignment matrices.	**OMoCap**: Vicon 8 cameras.
		Pelvis, thigh (external lateral), shin (anteromedial), and foot (dorsum).			
Allseits (B) et al. [58]	4	MAPRs.	**STP**: Step length, stride length, and speed.	**SP**: Local minimum of the shin angular velocity (HS). **MB**: It employs an inverted pendulum model algorithm considering segment lengths.	MatScan walkway (MA, USA).
		Thighs and shins.			
Botzel et al. [59]	4 and 5	IDG500, CA, USA, and ADXL335, MA, USA.	**GP**: Stance phase, swing phase, double support time, HS and TO events. **STP**: Stride time and cadence, separation of feet, and step length.	**OE**: Kalman filter. **SP**: Peaks and valleys of the gyroscope signal to obtain HS and TO. **MB**: Two models—comprising four and five segments—were created, incorporating the pelvis.	**OMoCap**: Oqus Qualisys 8 cameras.
		Shins and thighs; in the case of 5 sensors, a pelvic segment is added.			
Niswander (A) et al. [60]	5	MTw, Xsens.	**GP**: Support phase time and swing phase time, HS and TO events.	**SP**: Velocity minima and zero-crossings in the mediolateral direction; acceleration peaks in the craniocaudal and anteroposterior directions to obtain HS and TO.	Instrumented mat. Protokinetics LLC (PA, USA).
		Heel, dorsum of the foot, lateral distal third of the tibia, lateral middle third of the tibia, and medial proximal third of the tibia.			
Yeo et al. [61]	5	BioSensics LEGSys+, MA, USA.	**STP**: stride length and time, step length, and cadence. **JA**: Knee and hip (Range of Motion).	**Proprietary method**: LEGSys software (BioSensics, Cambridge, MA, USA) to obtain metrics.	**OMoCap**: Qualisys 6 cameras.
		Anterior aspect of the shins and thighs, and the center of the posterosuperior iliac crests.			
Allseits © et al. [62]	5	Multi-Axial Profile Recorder.	**GP**: Gait cycle time, single limb support time and double limb support time, HS and TO events.	**SP**: Trough following the zero-crossing (HS) and zero-velocity instant (TO) on angular velocity.	Mat System walkway (MA, USA).
		Shins, thighs and sacrum.			
Lebel et al. [17]	6	IGS-180 system, Synertial, Sussex, United Kingdom.	**JA**: Ankle (dorsiflexion/plantarflexion, eversion/inversion), knee (flexion/extension), hip (abduction/adduction, internal/external rotation, flexion/extension), and pelvis (in transverse, frontal and sagittal planes).	**Not Reported**: Algorithms not specified by authors.	**OMoCap**: Vicon 12 cameras.
		Left foot (dorsal), left tibia (lateral), left femur (lateral), pelvis (L5), upper back (T2), and head.			
Luo et al. [63]	6	Dot, Xsens .	**GP**: Stance time, swing time, and double support time. **STP**: Stride length, stride time, speed, step time, and cadence. **JA**: Ankle (dorsiflexion/plantarflexion).	**SP**: Sagittal plane angular velocity (zero-crossing for HS, negative peak for TO, positive peak for mid stance).	**OMoCap**: Vicon 13 cameras.
		In the Footwear (gap in the insole), dorsum and heel.			
Potter et al. [14]	7	Opal, APDM.	**STP**: Stride length and step width. **JA**: Ankle, knee and hip (in transverse, frontal and sagittal planes for all 3 joints).	**OE**: 9-DoF ErKF (Error-State Kalman Filter). **CC**: Use of ZUPT (Zero Velocity Update), correction of gravitational tilt, joint center, and joint axis to correct the measurements provided by the ErKF.	**OMoCap**: Vicon 19 cameras.
		Feet (dorsal), shins (medial), femurs (lateral), and pelvis.			
Teufl et al. [64]	7	MTW , Xsens.	**GP**: Support phase time, swing phase time, single-support time, and double-support time. **STP**: Stride length, step length, step width, swing width, step time, stride time, cadence, and speed.	**OE**: Sensor fusion algorithm (It is not specified which one). **SP**: Detection of the maximum distance traveled by the heel in the direction of progression relative to the pelvis to segment the gait cycle.	**OMoCap**: OptiTrack Motive (OR, USA).
		Feet (dorsal), shins (lateral), femurs (lateral), and pelvis.			
McGrath (B) et al. [65]	7	Opal, APDM.	**JA**: Knee (flexion/extension) and hip (internal/external rotation, abduction/adduction and flexion/extension).	**OE**: Joint angles were estimated using the Levenberg–Marquardt algorithm. **MB**: anatomical constraints and joint models to improve angle estimation.	**OMoCap**: Vicon 13-camera system.
		Feet, shins, thighs, and pelvis.			
Rekant et al. [66]	7	MyoMotion, Noraxon, AZ, USA.	**JA**: Ankle (dorsiflexion, abduction, and rotation), knee (flexion), and hip (flexion, abduction, and rotation).	**Proprietary method**: Company’s proprietary software to obtain metrics.	**OMoCap**: Vicon 14 cameras.
		Feet (dorsal), shins (lateral), femurs (lateral), and pelvis.			
Lebleu et al. [67]	7	x-IMU, x-io Technologies, Bristol, United Kingdom.	**JA**: Ankle, knee, hip, and pelvis angles (in transverse, frontal and sagittal planes for all 4 joints)	**OE**: Attitude and Heading Reference System (AHRS) by Mahony. **CC**: Alignment matrices derived from four functional calibration methods based on specific active joint movements.	**OMoCap**: Vicon 8-camera system.
		Feet (dorsal), shins (lateral), femurs (lateral), and pelvis (L5).			
Shuai et al. [68]	7	The Perception Neuron system, Beijing, China.	**JA**: Ankle, knee, and hip (in transverse, frontal and sagittal planes for all 3 joints).	**Proprietary method**: Kalman filter to obtain orientation (performed by Axis Studio software (NOITOM, Beijing, China)).	**OMoCap**: OptiTrack Prime17W with 8 cameras.
		Feet (dorsal), shins (lateral), femurs (lateral), and sacrum.			
Cho et al. [69]	7	MPU-9250	**GP**: Percentage of stance phase and percentage of swing phase. **STP**: Stride length and walking speed. **JA**: Ankle, knee, and hip (in transverse, frontal and sagittal planes for all 3 joints).	**OE**: AHRS module for obtaining orientation and, subsequently, angles. **SP**: First derivative of foot Euler angles for event detection; double integration of acceleration for spatial parameters.	**OMoCap**: Vicon Mx-T10 and Raptor-E Digital Real-Time System (CA, USA).
		Feet (dorsal), shins (anterior), thighs (anterior), and lower abdomen.			
Al-Amri et al. [70]	7	MTw2, Xsens.	**JA**: Ankle, knee, and hip (in transverse, frontal and sagittal planes for all 3 joints).	**Proprietary method**: Joint angles were obtained using MVN BIOMECH software (Version 4.4).	**OMoCap**: Vicon 10 cameras.
		Feet (dorsal), shins (medial), femurs (lateral), and sacrum.			
Descollonges et al. [71]	7	NeuroSkin’s System, Lyon, France.	**STP**: Step length, stride length, and speed.	**SP**: Distances traveled were calculated via double integration of vertical hip acceleration. **MB**: For spatial parameters, the inverted pendulum method was used.	GAITRite mat.
		Lumbar spine, femurs, tibias, and feet (dorsal).			
Dorschky et al. [72]	7	Custom sensors, GmbH, Erlangen, Germany.	**JA**: Ankle, knee, and hip (flexion/extension for all 3 joints).	**Not Reported**: Algorithms for obtaining the orientation not specified by the authors. **MB**: Using a musculoskeletal model and gradient-based optimal control to obtain joint angles from the orientation.	**OMoCap**: Vicon 16 cameras + force plate, Kistler Instruments.
		Lower back, thighs (external lateral), shins (external lateral), and feet (midfoot).			
Berner et al. [73]	7	myoMOTION Research Pro.	**JA**: Pelvis (tilt, obliquity and rotation), hip (flexion/extension, adduction/abduction, external/internal rotation), knee (flexion/extension), and ankle (dorsiflexion/plantarflexion).	**Proprietary method**: HS and TO events obtained using system’s software and Kalman filter programmed at the factory to obtain orientation.	**OMoCap**: MX T-series Vicon 8 cameras + Bertec force platforms (OH, USA).
		Feet (dorsum and proximal to the equinus break), shins (anteromedial), thighs (lateral) and pelvis.			
Šlajpah et al. [74]	7	Not reported.	**JA**: Ankle, knee, and hip (flexion/extension for all 3 joints).	**OE**: An Extended Kalman Filter (EKF) was used, and the orientation was expressed in quaternions.	**OMoCap**: Optotrack + 2 instrumented insole inserts Parotec-System, Poromed GmbH.
		Feet (external lateral), shins (external lateral), thighs (external lateral) and lower back.			
Bailey et al. [75]	8	Dot, Xsens.	**JA**: Ankle, knee, hip, pelvis, and trunk (in transverse, frontal, and sagittal planes for all joints).	**OE**: Use of Madgwick’s magnetometer-free algorithm to obtain sensor orientations.	**OMoCap**: Vicon 11 cameras.
		Feet (dorsal), shins (anterior), femurs (anterior), pelvis, and trunk (at the level of the sternum).			
Adamowicz et al. [76]	8	Opal v2, APDM.	**JA**: Hip (flexion/extension, adduction/abduction, and internal/external rotation).	**OE**: Obtained using Kalman filter. **CC**: Sensor-to-segment alignment using kinematic constraints and a static pose.	**OMoCap**: Vicon 19 cameras.
		Feet (dorsal), shins (medial), femurs (lateral), lumbar, and chest.			
Niswander (B) et al. [77]	10	MTw, Xsens.	**JA**: Ankle, knee, and hip (flexion/extension for all 3 joints).	**Proprietary method**: Quaternion-based thigh orientation obtained using Xsens. **CC:** Static calibration defining craniocaudal (stationary vector) and mediolateral (cross product) orthogonal axes.	**OMoCap**: Vicon 12-camera system.
		Right leg: Foot (heel and dorsum), right tibia (2 lateral and 1 medial), right thigh (1 anterior, 2 laterals, and 1 posterior), and torso (sacrum and L4-L5).			
Piche et al. [78]	11	iSen STT Systems, San Sebastian, Spain.	**JA**: Ankle, knee, and hip (flexion/extension for all 3 joints).	**Proprietary method**: Kinematics generated by iSen’s proprietary software v2022.0.	**OMoCap**: OptiTrack Motive 9 cameras + force plates on the treadmill.
		Feet (heel and dorsum), shins (frontal), femurs (external lateral), pelvis, upper back, and chest.			
Slade et al. [79]	14	Commercial IMUs ISM330DHCX, Adafruit, in-house development acquisition system, NY, USA.	**JA**: Ankle (dorsiflexion/plantarflexion), knee (flexion/extension), hip (flexion/extension, abduction/adduction and rotation).	**OE**: Use of the Mahony Filter algorithm. **MB**: Inverse kinematics to obtain angles is performed using OpenSim software (Version 4.2.).	**OMoCap**: OptiTrack.
		Feet (dorsal), shins (external lateral), femurs (external lateral), pelvis, torso (T2), hands (external lateral), forearms (external lateral), and upper arms (external lateral).			
Fan et al. [80]	17	HWT901B, Wit Motion, Shenzhen, China.	**GP**: Gait cycle time, maximum foot clearance, HS and TO events, **STP**: Step length.	**OE**: Using a Two-step complementary filter. **SP**: Negative peak value of foot’s angular velocity to obtain HS and TO.	**OMoCap**: NOKOV 12 cameras (Beijing, China).
		Feet, Shins, thighs, shoulders, upper arms, fore arms, hands, head, spine and pelvis.			

To make the table easier to read, acronyms are bolded: **: IMU**: Inertial Measurement Unit; **STP**: Spatiotemporal Parameters; **GP**: Gait Phases and Events; **JA**: Joint Angles; **HS**: Heel Strike; **TO**: Toe-Off. **OMoCap**: Optical Motion Capture System. **OE**: Orientation Estimation; **SP**: Signal Processing; **CC**: Calibration and Correction; **MB**: Model-Based Methods.

## Data Availability

No new data were created or analyzed in this study. Data sharing is not applicable to this article.
